# Wirelessly Observed Therapy to Optimize Adherence and Target Interventions for Oral Hepatitis C Treatment: Observational Pilot Study

**DOI:** 10.2196/15532

**Published:** 2020-04-30

**Authors:** Maurizio Bonacini, Yoona Kim, Caroline Pitney, Lee McKoin, Melody Tran, Charles Landis

**Affiliations:** 1 Mission Gastroenterology and Hepatology San Francisco, CA United States; 2 Proteus Digital Health Redwood City, CA United States; 3 Paul G Allen School of Computer Science and Engineering University of Washington Seattle, WA United States

**Keywords:** chronic hepatitis C, HCV, compliance, sustained virologic response, antivirals, sofosbuvir

## Abstract

**Background:**

A fixed-dose combination of ledipasvir/sofosbuvir (LDV/SOF) is efficacious in treating chronic hepatitis C virus (HCV) infection; however, objective adherence to prescribed regimens in real-world clinical settings has not been well studied.

**Objective:**

This study aimed to evaluate adherence and virologic outcomes in patients with chronic HCV infection treated with LDV/SOF using a novel digital medicine program that directly measures drug ingestion adherence.

**Methods:**

This prospective, observational, open-label, single-arm pilot study was conducted at 2 clinical research sites and followed patients with HCV infection who were prescribed LDV/SOF along with an ingestible sensor. Patients were treated for 8 or 12 weeks. The main outcomes were ingestion adherence, medical interventions, virologic response, safety, and patient satisfaction.

**Results:**

Of the 28 patients (mean 59 years, SD 7), 61% (17/28) were male, 61% (17/28) were non-Caucasian, and 93% (26/28) were treatment naïve. All 28 had genotype 1 HCV, and of these, 27 completed an 8- or 12-week treatment. Patients used the digital medicine program for 92% of the expected days; the overall mean ingestion adherence rate was 97%. Providers used the digital medicine program data for same-day medication therapy management in 39% (11/28) of patients. End-of-treatment response was achieved in all the available 21 of 28 patients. Sustained virologic response at 12 weeks or more was achieved in 26 of 28 patients; of the 2 patients who relapsed, one had less than 90% adherence and the other had greater than or equal to 95% adherence, lending insights into reasons for treatment failure. A total of 4 subjects reported nonserious adverse events, which were resolved.

**Conclusions:**

The findings of this study suggest that digital medicines can be used for wirelessly observed therapy to support adherence to antiviral HCV therapy, reduce unnecessary medication wastage and retreatment costs, and potentially optimize sustained virologic response rates, especially in populations at high risk for nonadherence.

## Introduction

### Background

The World Health Organization estimated that in 2015, 71 million people worldwide were living with chronic hepatitis C virus (HCV) infection. Although recent studies report that the incidence of HCV has decreased during the past 50 years [1-3], estimates obtained from modeling suggest that in 2015, there were still 1.75 million new HCV infections worldwide—a global incidence rate of 23.7 per 100,000 [4]. It is estimated that up to 20% to 30% of individuals with chronic HCV infection will develop liver cirrhosis with associated complications, including hepatocellular carcinoma, liver decompensation, and increased mortality [4,5]. In addition, there has been a recent steep increase in HCV diagnoses in the United States, owing, in part, to the opioid epidemic [6]. Treatment for chronic HCV infection has evolved rapidly over the past 5 years, with new all-oral, single-tablet, direct-acting antiviral (DAA) agents. Among the currently available medications is a once-daily, fixed-dose combination of ledipasvir and sofosbuvir (LDV/SOF) for the treatment of chronic HCV genotype 1 (GT-1) infection [7].

A recent clinical trial investigating the efficacy of an LDV/SOF combined formulation, conducted in a real-world setting, showed high response rates in the per-protocol population following 8 weeks (98.2%) and 12 weeks (98.3%) but notably lower response rates in the intent-to-treat (ITT) population following 8 weeks (84.6%) and 12 weeks (85.1%) [8]. The differences between per-protocol and ITT outcomes highlight the importance of adherence and patient engagement to HCV therapy as an essential element in achieving sustained virologic response (SVR) or cure. In addition, in special populations, such as people who inject drugs, SVR rates are lower and are associated with better compliance [9].

A novel digital medicine program, Proteus Discover (Proteus Digital Health), evaluates medication adherence through wirelessly observed therapy and addresses these limitations [10]. The digital medicine program directly measures medication ingestion adherence, heart rate, physical activity, and other biometrics. It then provides real-time feedback to patients and health care providers via mobile devices and a dedicated Web portal to support patient self-management and facilitate therapy optimization by the provider.

### Objective

This study aimed to evaluate medication ingestion adherence and virologic outcomes in patients with chronic HCV infection who were prescribed LDV/SOF along with the digital medicine program for wirelessly observed therapy. Safety and patient satisfaction related to digital medicine program were also assessed.

## Methods

### Study Design

This was a 24-week, single-arm, prospective, open-label, pilot study designed to assess real-world adherence to LDV/SOF treatment among patients with HCV infection using the digital medicine program. The study was conducted at 2 US clinical research sites from August 25, 2015, to November 9, 2017. Potential patients who met the inclusion criteria were identified through electronic medical records and contacted to assess their interest in study participation.

An independent investigational review board (E&I) approved the study protocol*.* All aspects of this study were conducted in accordance with the US Food and Drug Administration (FDA) regulations, the International Council for Harmonisation E6 (R1) guideline for Good Clinical Practice, and applicable local, state, and federal laws.

### Patients

The study enrolled adult patients diagnosed with chronic HCV infection to be initiated on fixed-dose LDV/SOF. Patients were included if they had HCV viremia greater than 50,000 IU/mL, capacity to use a smartphone or tablet (assessed by the investigators), and adequate data connectivity at home via cellular service or a secure wireless internet network. Patients were excluded from the study if any of the following criteria were met: history of skin sensitivity to adhesives, history of acute or chronic dermatitis, decompensated cirrhosis, liver transplant candidate, lack of insurance coverage for fixed-dose LDV/SOF, BMI greater than 40 kg/m^2^, and currently known to be pregnant or nursing an infant. For women of childbearing potential, the following exclusion criteria were considered: not using an acceptable form of contraception for at least 3 months before the start of the study and throughout the study, lactating, current participation in another clinical trial, terminal illness (≤1 year of life anticipated), inability to swallow pills, or any condition that in the investigator’s opinion could preclude safe participation in the study. All patients signed a written informed consent form before entering the study.

### Digital Medicine Program

The digital medicine program consists of 3 components that function together: (1) LDV/SOF that was individually repackaged with FDA-approved ingestible sensors by a specialty pharmacy; (2) an FDA-approved wearable sensor patch, which is worn by the patient over the left upper quadrant and collects time-stamped medication ingestion events and physiologic metrics (eg, steps, activity, rest, and heart rate); (3) a mobile app and software that calculates and summarizes adherence patterns, physical activity, rest, and other self-entered clinical data that patients can view through a mobile device and providers can view through a secure Web portal. After being swallowed, the ingestible sensor activates after 2 to 3 min, sending a brief signal to the patch before passing through the body naturally. The digital medicine program directly measures medication ingestion adherence, providing objective data to patients and providers.

### Procedures

At the screening visit, patients’ medical records were reassessed to confirm eligibility, baseline laboratory values were taken (per usual care), signed informed consent was obtained, and a urine sample for pregnancy testing was collected. Patients received hands-on training on using the digital medicine program and were instructed to change the sensor patch every 7 days or sooner, as determined by the sensor. Patients were taught to review the app daily to obtain feedback on their medication adherence. Patients used the digital medicine program for the duration of their 8- or 12-week treatment period. The determination of treatment duration was based on the patient’s baseline liver severity and HCV viral load as well as prior therapy.

Throughout the treatment period, a provider reviewed patients’ adherence data from a secure Web portal at their discretion. If a missed dose was detected, the patient was contacted by phone to account for the missing dose and was provided adherence counseling if needed. Nonadherent patients were asked to return to the clinic at weeks 4 or 6 for laboratory assessment and adherence counseling. Patients with high levels of adherence were not required to return to the clinic unless the provider wanted to assess other laboratory examinations, such as liver function tests. HCV viral load was assessed at the end of the treatment (8 or 12 weeks, depending on patient treatment plan) and at 12 weeks or more after the treatment ended. Patients also completed a satisfaction survey at the end of the treatment. All clinic procedures were performed per usual care except for the digital medicine program intervention, which prompted adherence interventions. Any technical issues were handled by Proteus Digital Health, and clinical issues were forwarded to the site for evaluation.

### Outcomes

The primary end point of the study was the percentage of subjects with greater than or equal to 95% medication ingestion adherence. Ingestion nonadherence was defined as a failure to trigger iPad (Apple Inc) activation within the 4-hour window surrounding the scheduled dose, that is, the number of medication ingestions measured by the digital medicine program plus same-day patient confirmation of ingestions reported to the health care provider over the total expected number of ingestions during the treatment period. Other outcomes included virologic response, medical interventions made, safety of the digital medicine program, and patient satisfaction.

### Statistical Analysis

Owing to the pilot nature of the study, a priori sample sizes were determined. All analyses were performed on the ITT population, defined as all patients who received at least one dose of the study medication. All end points were presented descriptively. Categorical variables were described as proportions, whereas continuous variables were described as mean (SD) or median (IQR) where appropriate. Multivariate linear regressions were conducted and used to assess demographic, clinical, and behavioral predictors of adherence using R version 3.5.1 (The R Foundation for Statistical Computing).

## Results

### Treatment Cohort

A total of 31 patients were screened, and of these patients, 28 met the inclusion criteria and were enrolled in the study (ITT population). All 28 patients (mean 59 years, SD 7; 61% [17/28] male; 61% [17/28] non-Caucasians; 93% [26/28] treatment naïve) had HCV GT-1. Moreover, 1 patient withdrew from the study 21 days after initiating digital medicine program use, and 27 patients completed digital medicine program use for 8 weeks (n=10) or 12 weeks (n=17) as prescribed. In addition, 13 (46%) patients had psychiatric comorbidities, and 9 (32%) of the patients had a history of drug abuse. Table 1 presents the baseline characteristics of enrolled patients.

**Table 1:** Baseline demographics and characteristics (N=28).

Characteristic		Value
Male, n (%)		17 (61)
Age (years), mean (SD)		59 (7)
**Genotype, n (%)**		
	1a	25 (89)
	1b	3 (11)
**Race, n (%)**		
	Caucasian	11 (39)
	African American	10 (36)
	Hispanic/Latino	4 (14)
	American Indian/Alaska Native and other	3 (11)
Cirrhosis, n (%)		1 (4)
**Prior treatment history, n (%)**		
	Treatment naïve	26 (93)
	Previous treatment relapse	2 (7)
**Frequency of mobile phone use per week, n (%)**		
	<1 time	3 (11)
	2-4 times	4 (14)
	5-7 times	21 (75)
**Education level, n (%)**		
	Less than high school	2 (7)
	Some high school or high school graduate	12 (43)
	Some college or college graduate	13 (46)
	Postgraduate education	1 (4)
**Income level (US $/year), n (%)**		
	<25,000	23 (82)
	≥25,000	5 (18)
Psychiatric comorbidity, n (%)		13 (46)
Prior or current drug abuse history, n (%)		9 (32)

### Adherence Outcomes

High rates of medication ingestion adherence were observed, with 89% of patients achieving greater than or equal to 95% adherence. Patients were connected to the digital medicine program for 92% of expected days, with an overall mean adherence rate of 97%. Using a stepwise approach to select variables to include in the model, multivariate linear regression showed that being African American, having high school education or less, and having a psychiatric comorbidity were found to be consistently significant predictors of nonadherence (Table 2). Mean adherence was lower in African Americans than others (88% vs 96%), those with high school education vs those with less (91% vs 96%), and individuals with a psychiatric comorbidity vs those without a psychiatric comorbidity (90% vs 96%). There was no difference in adherence between patients treated for 8 weeks vs 12 weeks.

**Table 2:** Predictors of adherence using stepwise multivariate linear regression.

Factors	Model coefficient^a^
Race**⸺**African American vs other	−8.8
Education**⸺**high school graduate or less	−5.7
Comorbidities**⸺**psychiatric comorbidity	−5.4

^a^Only statistically significant factors (*P*<.05) are included.

### Efficacy Outcomes

Virologic results at the end of the treatment were available for 21 patients, all of whom achieved an end-of-treatment response. All 28 subjects were assessed at 12 weeks or more posttreatment. Among those subjects, 26 (93%) achieved SVR, including 2 treatment-experienced patients who had previously failed treatment. Of the 2 subjects who did not achieve SVR, one had documented suboptimal adherence (<90%) and the other had greater than or equal to 95% adherence, suggesting viral resistance as the cause of relapse.

### Provider Medication Interventions Made

Health care providers used the digital medicine program data for timely (same-day) assessment of medication ingestion adherence. A total of 130 documented initial missed doses occurred among 11 (39%) patients. Interventions made to mitigate nonadherence among these patients included 75 follow-up phone calls, 21 adherence counseling events, and 5 follow-up visits; some patients required multiple interventions per missed dose. No action was performed for the remaining 35 events (eg, patient unreachable).

### Patient Satisfaction

**Figure 1:**
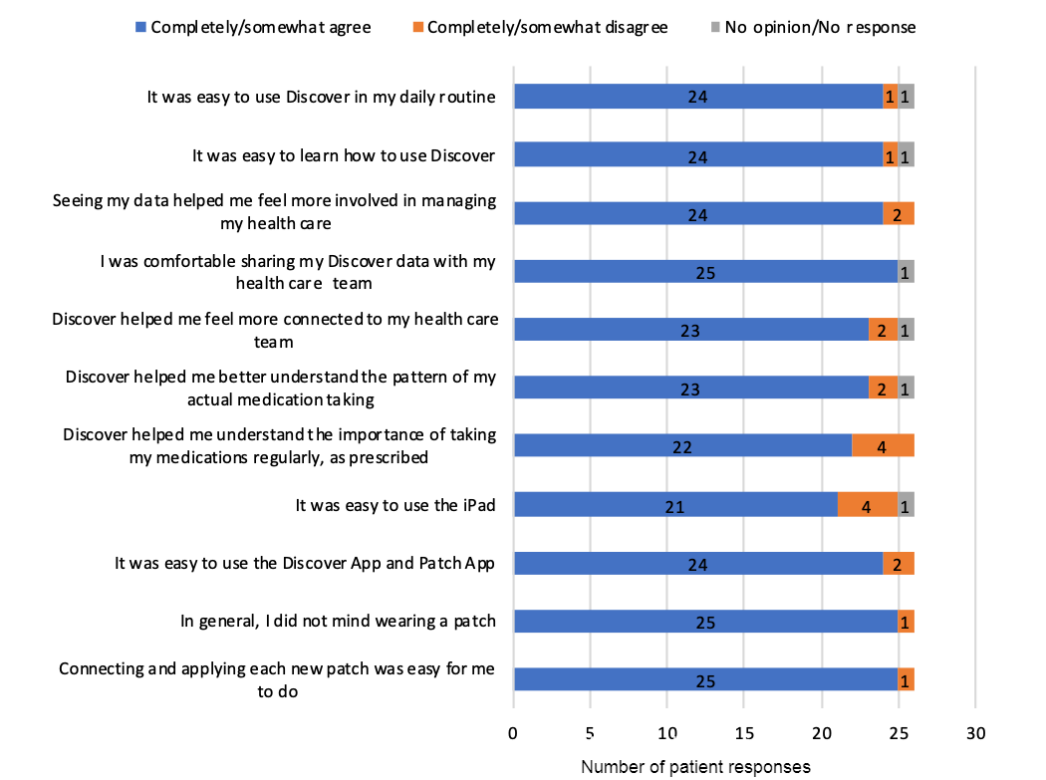
Patient satisfaction survey results (n=26).

Overall satisfaction with the use of the device was high among study subjects (Figure 1). Most (92%) patients completely or somewhat agreed that the DMP was easy to use in their daily routine and helped them feel more involved in managing their health care, 96% did not mind wearing the patch and sharing their data with their health care team, and 85% agreed that the DMP helped them understand the importance of taking their medications regularly.

### Safety Findings

A total of 4 nonserious, device-related adverse events were reported in 4 subjects during the study. These adverse events were reflective of commonly reported symptoms of irritant contact dermatitis (rash). Of these, 1 reported adverse event (itching) was assessed by the investigator as severe and resolved without any action taken with the DMP device. The remaining 3 reported adverse events of rash were assessed by the investigator as mild in severity and resolved.

## Discussion

### Principal Findings

This real-world pilot study in a cohort of patients with chronic HCV infection demonstrated that the use of the DMP with same-day interventions (when needed) prompted high rates of medication adherence (97%), despite the presence of multiple risk factors for nonadherence, such as race, psychiatric comorbidities, and substance use or abuse. More importantly, SVR at 12 weeks or more was achieved in 92.8% (26/28) of the patients. Patients were connected to the DMP 92% of the time, indicating high patient engagement, and the system was easy to use even in those patients with historically low mobile phone use. In addition, the DMP had a favorable safety profile and provided data to facilitate timely adherence interventions. Thus, the unique ability of the DMP to provide objective adherence data enabled timely medication interventions aimed at improving adherence and optimizing outcomes, even in high-risk patients who have previously failed therapy.

Large clinical trials have demonstrated that treatment with once-daily, combined, fixed-dose LDV/SOF therapy is effective in achieving SVR in a vast majority of patients with chronic HCV infection [8,11-13]. However, adherence to prescribed treatment regimens is critical to achieving treatment success [14]. Nonadherence to HCV DAA regimens has been shown to be associated with a lower rate of SVR [12], particularly in patients with psychiatric illness [15]. Current indirect assessment methods (eg, medication possession ratios, patient self-report, pill counts, and electronic pill bottles) may overestimate actual medication adherence [16]. In contrast, DMP addresses the inherent limitations of these methods through direct objective measurement of medication ingestion. In addition, and perhaps more importantly, it allows real-time digital intervention by patients and/or clinicians when doses are not recorded as *ingested*.

High levels of adherence have been reported in large clinical trials using various measurement assessments, including electronic medication event monitoring (MEM) caps, pill counts, and patient reports [12,13]. However, the accuracy of the current measurement methods may be questionable because of the limitations of each. For example, patients may purposefully mislead the MEM system by accidentally actuating the container without taking the medication [17]. Intentional manipulation of pill counts (*pill dumping*) and falsified patient reports to appear adherent must also be considered when measuring medication adherence [17]. None of these methods allow the patient or provider an opportunity to respond quickly or proactively to perceived missed doses [18,19]. Although measurement of the proportion of days covered is commonly used to assess long-term adherence in patients with chronic illnesses [20], the use of prescription refill data does not allow for timely detection of nonadherence, and it does not necessarily indicate that all the medications have been taken [21].

Medication adherence is of particular concern among patients with HCV treated with fixed-dose LDV/SOF therapy, many of whom are at high risk for nonadherence because of transient living situations, depression, neurocognitive impairment, psychiatric comorbidities, and concurrent alcohol and other substance abuse [22,23]. As successful treatment is dependent on strict adherence to the prescribed medication regimen, nonadherence may result in the same poor clinical outcomes (eg, liver cirrhosis, hepatocellular carcinoma, liver decompensation, and increased mortality) associated with nontreatment [4,5].

The DMP has been studied in multiple therapeutic areas, including cardiovascular and infectious diseases. In a randomized controlled trial of the DMP compared with usual care in patients with hypertension and diabetes, those using the DMP with their prescribed medications experienced greater reductions in systolic blood pressure (BP; mean change −24.6 vs −15.2; mean difference −9.4; SE 2.7; 95% CI −14.6 to −4.2 mm Hg) and glycohemoglobin (mean change −0.08% vs 0.28%; mean difference −0.57%; 95% CI −1.53 to 0.39) at 12 weeks [24]. In addition, a significantly higher number of subjects reached their BP goal in the DMP group compared with those in usual care after 12 weeks (98% vs 52%; mean difference 46%; 95% CI 7.1% to 84.5%). The DMP was also studied as wirelessly observed therapy in the setting of tuberculosis treatment, demonstrating a positive detection accuracy for wirelessly observed therapy of 98.4% (95% CI 97.5% to 99%) and confirming 54% more doses than even the previous gold standard of directly observed therapy [25]. The accuracy and objectivity of this DMP at quantifying and recording medication adherence have been firmly established in the literature [26].

Our results are similar to those reported in recent adherence and efficacy studies [8,11-13]. However, deriving correlations between adherence and treatment effect is problematic. For example, Petersen et al [13] reported 97.6% adherence as measured by an MEM system but provided no data regarding treatment effect. Conversely, neither of the 2 efficacy studies reported medication adherence data [8,11]. In addition, a recent study highlighted the ineffectiveness of low-cost reminder devices in improving adherence among nonadherent patients with chronic conditions, suggesting the need for objectivity, effectual interventions, and personalized feedback [27].

### Strengths and Limitations

A key strength of our study was the ability to directly measure medication ingestion, providing objective adherence data to better inform medical decision making. Although the small study population does not allow for definitive assessment of correlations between adherence and treatment effect, positive associations can be inferred from the timely interventions on missed doses, thereby improving adherence, which is expected to result in SVR. By extension, another positive point is the ability to improve patient care by contacting patients in real time and proactively assisting with missed doses.

Our study is limited by the single-arm design that precludes direct comparison with a usual care group. Furthermore, because of the small sample size, our results may not accurately reflect the broader population of patients with chronic HCV infection [28]. A larger multicenter clinical trial is currently underway to confirm and further elucidate these findings.

As reported by Moreno et al [29], improving patient access to HCV treatment will likely yield significant cost reductions for payers, accrued from the long-term reduction in prevalent and incident cases, mortality, and medical costs. Technology such as DMP could ensure that more patients with risk factors for nonadherence can complete therapy to ensure maximal real-world SVR rates. As reported in this study, although 6 patients failed to attend the end-of-treatment assessment for unknown reasons, all 28 patients were assessed at the 12-week or more evaluation period, where 26 (93%) achieved SVR. Patients were connected to the digital medicine program 92% of the expected days, indicating high patient engagement, and the system was easy to use even in those patients with historically low mobile phone use. In addition, the digital medicine program had a favorable safety profile and provided data to facilitate timely adherence interventions.

### Conclusions

These data suggest that the digital medicine program can support adherence to therapy, enhance patient engagement, reduce unnecessary medication wastage, and optimize SVR rates in patients with chronic HCV infection, including those with multiple risk factors for nonadherence and in those who have previously failed therapy.

## Abbreviations

BP: blood pressure
DAA: direct-acting antiviral
FDA: Food and Drug Administration
GT-1: genotype 1
HCV: hepatitis C virus
ITT: intent-to-treat
LDV/SOF: ledipasvir/sofosbuvir
MEM: medication event monitoring
SVR: sustained virologic response
